# Dual-mode sensing strategy for assaying phosphate ions using Fe, N-co-doped carbon dots with peroxidase mimetic activity

**DOI:** 10.1007/s00216-025-06279-z

**Published:** 2025-12-26

**Authors:** Shymaa S. Soliman, Amr M. Mahmoud, Amira M. Kessiba, Rasha M. Ahmed

**Affiliations:** 1 Analytical Chemistry Department, Faculty of Pharmacy, October 6 University, October 6 City, Giza 12858 Egypt; 2 Pharmaceutical Analytical Chemistry Department, Faculty of Pharmacy, Cairo University, El-Kasr-El Aini Street, Cairo, 11562 Egypt; 3 Pharmaceutical Chemistry Department, Faculty of Pharmacy, Misr International University, Misr-Ismailia Road, El Obour City, Cairo, 4650241 Egypt

**Keywords:** Nanozymes, Iron-doped nitrogen quantum dots (Fe@N-dCQDs), Phosphate determination, Peroxidase mimetic activity, Fluorescence quenching activity

## Abstract

Monitoring phosphate (PF) levels is critically significant because of its environmental, biomedical, and agricultural importance. In the current study, a novel dual-mode sensing platform was developed based on iron-doped nitrogen-rich carbon quantum dots (Fe@N-dCQDs), which exhibit both strong photoluminescence and intrinsic peroxidase mimetic activity. This dual property was the basis of PF determination, where its presence induced a concentration-dependent decrease in both colorimetric absorbance and fluorescence intensity, allowing for label-free and sensitive detection. The Fe@N-dCQDs were prepared using one-step pyrolysis and characterized by scanning electron microscope (SEM), transmission electron microscope (TEM), energy-dispersive X-ray spectroscopy (EDX), and Fourier transform infrared (FT-IR). The developed sensor showed excellent linearity in both detection modes (0.33–23.67 mM for the colorimetric method and 5.0–50.0 mM for the fluorometric method) with low detection limits (0.13 mM and 1.72 mM) and high correlation coefficients (0.9947 and 0.9998). Moreover, the sensor exhibited remarkable selectivity against common interfering ions typically found in environmental matrices, including phosphatic fertilizers. This simple, rapid, and cost-effective sensing strategy provides a promising tool for PF determination in environmental and agricultural samples and establishes Fe@N-dCQDs as a versatile nanomaterial for dual-mode analytical applications.

**Graphical Abstract:**

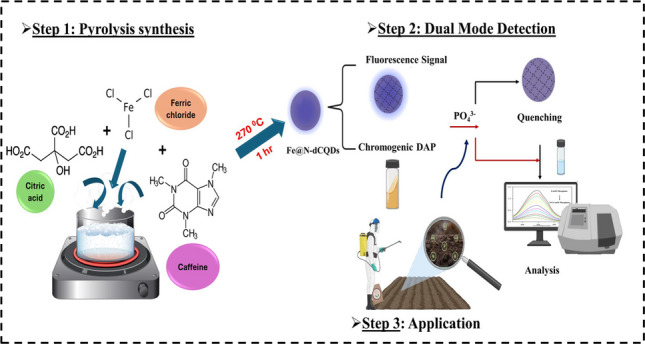

**Supplementary Information:**

The online version contains supplementary material available at 10.1007/s00216-025-06279-z.

## Introduction

Luminescent nanoparticles represent a rapidly growing field with diverse applications across several domains. They are widely utilized across different research areas, including analytical chemistry [1, 2], environmental science [3, 4], medicine [2, 5], energy [6, 7], agriculture [8, 9], and the pharmaceutical industry [10]. In biomedicine, they serve as potential alternatives to traditional fluorophores (dyes) that suffer from serious drawbacks, including photobleaching, instability, interference with biological processes, and toxicity [11, 12]. Their widespread popularity is mainly credited to their unique criteria, distinctive optical properties, and multifunctionality in several domains.

Carbon-based quantum dots (CQDs) are particularly notable as compelling and attractive semiconductor nanomaterials with remarkable optical and electronic properties [13, 14]. They outperformed traditional QDs in terms of good chemical inertness and solubility. They possess highly appealing photo-induced properties such as visible absorption, photoluminescence (PL), and photothermal effects. Their small size, excellent fluorescence, photostability, biocompatibility, ease of synthesis, and low toxicity have led to their application as sustainable and multifunctional fluorophores in numerous fields, such as bioimaging [15–17], sensing [18], optoelectronics [19], energy [20], and catalysis [21], besides being used as effective drug carriers.

Several methods, including hydrothermal synthesis, combustion oxidation, thermal decomposition, electrochemical synthesis, microwave irradiation, and pyrolysis of organic molecules, were reported for synthesizing CQDs [22–24]. However, these synthesis processes often result in CQDs with suboptimal optical properties [25, 26]; thus, preparing CQDs with higher quantum yield (QY) and multifunctionality is a controversial issue that needs to be pursued. Chemical doping with heteroatoms has emerged as an effective approach to modulate and enhance the photoluminescence performance of CQDs, thereby widening their potential applications [27, 28]. Nitrogen-doped CQDs (NCQDs) combine the outstanding advantages of CQDs with the exceptional properties of doping to overcome their inherent limitations [29], making them ideal for application in different areas such as catalysis, ion probes, and biological imaging [28]. This may be attributed to different reasons, including the similarity of nitrogen’s atomic size to that of carbon, nitrogen’s higher electronegativity, and the availability of excess electrons for carbon atoms’ bonding, leading to an upward shift in the Fermi level, which enables nitrogen to chelate with amino and carboxyl groups present on the CQDs surface [28, 29]. This not only alters their optical properties but also introduces active sites that provide CQDs with new functionalities, such as mimetic enzyme activity, which deeply widens the application scope of CQDs.

Additionally, metal-doped CQDs have attracted the attention of researchers everywhere as a promising nano-doping technique [30, 31]. Within this category, iron-carbon quantum dots (Fe-CQDs) represent a distinctive subclass of CQDs that incorporates both the optical and fluorescence features of traditional CQDs with the unique magnetic and catalytic properties of iron [32]. This combination permits Fe-CQDs to exceed the traditional constraints of CQDs by enhancing their utility through the integration of optical functionality, magnetic responsiveness, and enzyme-like catalytic capabilities, which enable multifunctional applications such as dual-modality biomedical imaging and catalysis for environmental and biochemical sensing [30, 33], thereby positioning Fe-CQDs as an advanced evolution of CQDs.

Phosphate (PF) is considered one of the most essential nutrients for various biological, geological, and industrial applications. It helps maintain human health and enhances plant productivity as a key component in energy transfer, enzymatic activities, and biochemical functions [34]. In humans, about 85% of PF is located in bones, and the rest is primarily located inside different cells, participating in maintaining the structural integrity of bones and teeth along with other biological processes, including energy metabolism, DNA and RNA synthesis, and cell function and signaling [35]. In plants, PF is crucial for energy transfer, photosynthesis, and other metabolic activities [36]. It contributes to the synthesis of plants’ nucleic acids and membranes, facilitating cell division and growth while supporting root development, flowering, and seed production, thus directly affecting crop yields and agricultural productivity. Therefore, imbalances in PF levels can have serious consequences and direct impacts not only on living organisms’ growth, development, and functioning but also on plants’ maturity and yields, causing urgent implementation of chemical phosphatic fertilizers to compensate for the PF shortage and get optimum yields [37]. However, the misuse of these fertilizers may lead to several environmental, agricultural, and health issues [38, 39].

Developing effective and reliable methods for accurately maintaining and monitoring PF levels is in great demand. Recently, PF has been mainly determined using different analytical methods, including colorimetric [40–43], electrochemical [44–46], fluorescence [47–49], and chromatographic methods [50]. Some of these methods, especially colorimetric assays, may involve the incorporation of advanced nanomaterials during the analysis to enhance detection efficiency and accuracy. In recent years, several detection systems based on using CQDs have been developed for assaying PF [51–53].

In the current study, a sensitive colorimetric and fluorometric dual signal assay was developed for the determination of PF in different environmental matrices, including agricultural soil, commercial fertilizers, and river water, using nitrogen-doped iron CQDs (Fe@N-dCQDs). Dual-signal mode is used not only to improve results’ accuracy and to avoid false positives but also to provide complementary features and enhance the detection performance of the developed method through the differences in detection ranges of the different output signals. The utilized Fe@N-dCQDs were synthesized using the one-step pyrolysis method by using caffeine and FeCl_3_ as precursors. Then, the resulting carbon dots were used to catalyze the oxidation of O-phenylenediamine (OPD) by hydrogen peroxide (H_2_O_2_) into a yellow product of oxidized OPD (OPD_ox_) using acetate buffer (pH 4.0), producing a distinctive absorption peak and fluorescence signal at 448.4 nm and 443.0 nm, respectively. Upon adding PF to the solution, a complex will be formed between PF and the Fe@N-dCQDs, hindering the Fe@N-dCQDs’ catalytic and fluorescent activity, which resulted in a decrease in the OPD oxidized product along with the absorption intensity value at 448.4 nm. Photoluminescent fluorescence and durable oxidase activities were successfully achieved for the synthesized Fe@N-dCQDs compared to the reported peroxidase one. This nanoprobe facilitated the visual and fluorescence determination of PF using the dual-channel detection strategy, providing vast developments in the design and application of multifunctional CDs.

## Experimental

### Instrumentation

A UV-visible Shimadzu dual-beam spectrophotometer, model 1800, was used for capturing the absorption spectra. A 1.0-cm quartz cell, 1.0-nm slit width, and 0.2-nm sample interval were used during the scanning. The spectra were interpreted using Shimadzu 2.32 software. Ultra-sonication was used for the full dispersion of CQDs, and a pH meter (model 3505, Stone, UK) was utilized for adjusting the experiment’s pH values.

Fluorescence spectra were recorded using a Shimadzu spectrofluorophotometer (model: RF 5301 PC, Japan), equipped with a 150-W Xenon lamp. Slit widths for both the excitation and emission monochromators were set at 5.0 nm. A 1.0-cm quartz cell was used. The computations were done using Shimadzu RFPC version 2.04 software.

### Chemicals and reagents

The utilized reagents and solvents are of high analytical grade. Ultrapure water was acquired from a Milli-Q system (Millipore Corporation, France). Standard caffeine, hydrogen peroxide (H_2_O_2_), and O-phenylenediamine (OPD) were purchased from Sigma-Aldrich (Steinheim, Germany). Glacial acetic acid, sodium hydroxide pellets, citric acid, sodium dihydrogen phosphate, ferric chloride (FeCl_3_), hydrochloric acid, trizma, and citrate buffer were obtained from Piochem (Cairo, Egypt). Surface agricultural soil (depth of 0–20 cm) was obtained from a crop-producing field in Giza Governorate, Egypt, representing typical Egyptian cultivated soil. A river water sample was collected from a local freshwater source (Giza, Egypt) and filtered through a 0.22-µm membrane to remove suspended matter. Various phosphatic fertilizers were also obtained from the local market in Egypt.

### Procedures

#### Synthesis of iron-doped quantum dots (Fe@N-dCQDs)

A one-step pyrolysis method was followed for the synthesis of the nanoprobe, where different weights of citric acid, caffeine, and FeCl_3_ were mixed and heated at 270 °C for 1 h. Then, the resulting product was dispersed in 50 mL of distilled water and centrifuged for 30 min to get rid of any unreacted precursors or larger aggregates. A 22-µm syringe filter was later used for nanoprobe filtration into a monodispersed colloidal solution. The latter was then stored at 4 °C for future application and characterization.

#### Characterization

The synthesized nanoprobe was characterized using JEOL JSM-7001F scanning electron microscopy (SEM) and transmission electron microscopy (TEM). TEM micrographs were obtained at an accelerating voltage of 200 kV using a high-resolution TEM (JEOL JEM-2100, Japan). SEM and TEM images were acquired to examine the apparent morphology, particle shape, and size of the nanoprobe. Selected area electron diffraction (SAED) patterns were recorded to evaluate the crystallinity and phase composition of the QDs. Energy-dispersive X-ray spectroscopy (EDX) technique was carried out to examine the apparent elemental composition and purity of the synthesized QDs. A single-beam Shimadzu FT-IR instrument (Kyoto, Japan) with a high-energy light source and a KBr beam splitter coated with germanium was used to study chemical species interactions and composition changes.

#### Studying the peroxidase-like activity of Fe@N-dCQDs

UV-vis spectrophotometry was used to study the peroxidase-like activity of the prepared QDs by evaluating the catalytic oxidation of OPD substrate by H_2_O_2_ using an acetate buffer of pH 4.0. A mixture of 117.0 mM OPD, 3.8 mM H_2_O_2_, 200.0 mM acetate buffer (pH 4.0), and 1.0 mg mL^−1^ Fe@N-dCQDs was accurately prepared. A yellow-colored solution of OPD_ox_ was formed after 10 min in a 40 °C water bath and then scanned over a wavelength range of 300–600 nm against a blank experiment. The absorption intensity of the formed solution showed an increase, where the mimetic peroxidase activity of the Fe@N-dCQDs was detected at 448.4 nm.

#### Optimization of experimental conditions

##### Colorimetric determination

For spectrophotometry, various pH values of the acetate buffer (200 mM) were studied to select the optimal one that enhances the catalytic activity of the synthesized QDs. A 0.1 M HCl and 0.1 M NaOH were used for adjusting the pH values within a range of 2.0 to 8.0. The previous procedures were followed for the pH selection using fixed temperatures and concentrations.

Moreover, several temperatures were tried to choose the optimal one for experimentation. A water bath of different temperatures ranging from 40 to 80 °C was implemented during the study, while fixing other conditions as previously. Various solutions with different color intensities were obtained according to the employed temperature, where their visible spectra were noted and recorded for optimum parameter selection.

##### Fluorescence determination

The fluorescence intensity of the Fe@N-dCQDs was examined under different experimental conditions, such as medium pH, excitation light exposure, and ionic strength, to evaluate their stability and sensing capability. Various pH values were tried using 0.1 M HCl, 0.01 M citrate buffer, and 0.01 M trizma within a pH range from 2.0 to 11.0 to select the pH of maximum fluorescence intensity at fixed temperatures and concentrations. Photostability was assessed by continuously irradiating the Fe@N-dCQDs with a fluorescence lamp and monitoring the fluorescence intensity at 0, 1, 5, 10, 15, 30, 60, 90, and 120 min. In addition, the influence of ionic strength was examined by measuring the fluorescence intensity of the Fe@N-dCQDs in NaCl solutions of varying concentrations (0.2–1.5 M). The fluorescence quantum yield (QY) for Fe@N-dCQDs was measured using tryptophan as a reference standard (QY in trizma is 0.14) and calculated according to the following formula:

$$\varphi_x=\varphi_{ref}\;\left(\frac{I_x}{I_{ref}}\right)\;\left(\frac{A_{ref}}{A_x}\right)\;\;\left(\frac{\eta_x}{\eta_{ref}}\right)^2$$

where subscripts *x* and *ref* represent the Fe@N-dCQDs and tryptophan, respectively; *φ* is the quantum yield; *I* signifies the slope corresponding to the integrated fluorescence area and absorbance value, while *ƞ* is the refractive index of the solvent.

##### Construction of the calibration curve

Different concentrations of H_2_O_2_ were utilized to assess the OPD catalytic oxidation using the synthesized QDs, thereby evaluating the dynamic linearity range of the proposed method. A fixed OPD concentration of 117.0 mM and different concentrations of H_2_O_2_ ranging from 0.006 to 0.960 mM were used for the assessment. The previous procedures were followed, where the prepared concentrations were incubated in a 40 °C water bath for 10 min and then measured at 448.4 nm using the UV-vis spectrophotometer. A linear calibration curve was obtained within a concentration range of 0.006 to 0.887 mM for H_2_O_2_.

##### Steady-state kinetic measurements of the synthesized Fe@N-dCQDs

The peroxidase mimetic strength of the synthesized QDs was estimated by scanning the solutions’ kinetics against time. The procedures involved fixing the H_2_O_2_ concentration while varying the OPD concentrations. According to the substrate concentration, the absorbance rates were recorded every 10 min and monitored using Lambert-Beer’s law. The Michaelis-Menten equation, $\left(\frac1v\right)=\left(\frac{K_m}{V_{max}}\right)\;\left(\frac1{\left[S\right]+{\displaystyle\frac1{V_{max}}}}\right)$, was employed for calculating the relevant kinetic parameters by relating the Michaelis constant $\left(K_m\right)$ with the initial $V$ and maximum reaction rate (Vmax) along with the substrate concentrations $\left(S\right)$.

##### Detection of phosphate

The catalytic activity and fluorescence intensity of the synthesized Fe@N-dCQDs were exploited for PF detection. The latter was based on assessing the quenching effect of PF on the peroxidase mimetic activity and fluorescence nature of the synthesized QDs. For the colorimetric detection, a finely dispersed mixture of 117.0 mM OPD, 3.8 mM H_2_O_2_, 200.0 mM acetate buffer (pH 4.0), and 1.0 mg mL^−1^ Fe@N-dCQDs was prepared and mixed with different concentrations of 200 mM PF, then incubated in a water bath at 40 °C for 10 min. Afterward, the scanned absorption spectra were recorded over a wavelength of 300–600 nm, and the measurements were carried out at 448.4 nm using UV-vis spectrophotometry. On the other hand, the fluorescence assay of PF was performed by mixing the synthesized Fe@N-dCQDs with different concentrations of PF (ranging from 5.0 to 50.0 mM) and 0.01 M Trizma buffer (pH 10.5). Following excitation at 261.0 nm, the fluorescence intensity was measured at 443.0 nm. A calibration curve was constructed by plotting the quenching values against the different concentrations of PF, and a regression equation was computed.

A selectivity study was conducted where various interfering ions, such as Na^+^, NH_4_^+^, K^+^, SO_4_^2−^, Cl^−^, and NO_3_^−^ ions, were measured under the same conditions to assess their effects on the utilized reaction system compared to PF.

##### Detection of phosphate in different matrices

Surface agricultural soil (depth of 0–20 cm), river water samples, and commercially available fertilizers (labeled to contain 20% PF as P) were evaluated using the proposed Fe@N-dCQDs analytical assay. Soil samples were air-dried, gently ground, and sieved through a 2-mm mesh. The available PF was extracted according to the Olsen procedure [54, 55] using 0.5 M sodium bicarbonate solution (pH 8.5) and shaken for 30 min at room temperature, followed by filtration. The clear supernatant was collected and further diluted where necessary before the analysis. The river water was collected from a local water stream in Egypt, filtered through a 0.45-µm syringe filter to remove suspended solids, and analyzed without further pretreatment. For fertilizers, accurately weighed amounts of each product were dissolved in distilled water, filtered through a 0.45-µm membrane filter, and diluted appropriately before measurement. Afterward, the standard addition technique was applied to compensate for the potential matrix effects. Different concentrations of standard PF, ranging from 3.33 to 13.33 mM, were spiked into the reaction mixture. The quenching of the OPD oxidation signal was recorded, and the PF contents were determined and later validated against the ammonium molybdate reference method.

## Results and discussion

Quantum dots represent an innovative advancement in analytical chemistry, posing a blend of remarkable sensitivity, precision, and ecological sustainability. Their significant fluorescence enables the detection of different analytes in various matrices at low traces, making them appropriate for high-accuracy applications. They exhibit superior stability against several experimental conditions, including photobleaching and chemical degradation, which guarantee a consistent and efficient performance across multiple domains. Moreover, their non-toxicity, biocompatibility, and eco-friendliness properties lessen the environmental impacts of conventional methods. The current study emphasizes the pioneering application of Fe@N-dCQDs in assaying PF in different matrices, indicating their varied capability to provide fast, accurate, and reliable results, proving their wide applicability in real-life applications, including agriculture, while endorsing sustainable practices.

### Synthesis and characterization of the Fe@N-dCQDs

#### Synthesis of the Fe@N-dCQDs

A simple and accessible pyrolysis method was effectively used for synthesizing the established QDs. The method thermally breaks down carbon-rich precursors under controlled conditions. In the current study, citric acid, caffeine, and FeCl_3_ played a significant role in promoting pyrolysis, participating in customizing the structure and functionality of the QDs. These components synergistically collaborate to produce QDs with superior optical and magnetic properties, thereby increasing their effectiveness in various environmental and industrial applications. Citric acid, a carbon-rich precursor, provides the primary carbon framework for QDs while stabilizing the structure via its carboxylic groups. Caffeine offers nitrogen heteroatoms to enhance the QD optical properties and stability. Ferric chloride (FeCl_3_) acts as an iron source for effectively imparting the unique magnetic, catalytic, and sensing properties of iron into the developed QDs. This synergistic precursor design produced ultra-small, highly dispersed dots with a narrow size distribution and abundant surface functional groups (–NH, –OH, –COO⁻), while simultaneously incorporating iron and nitrogen to generate Fe–N coordination sites.

**Scheme 1:**
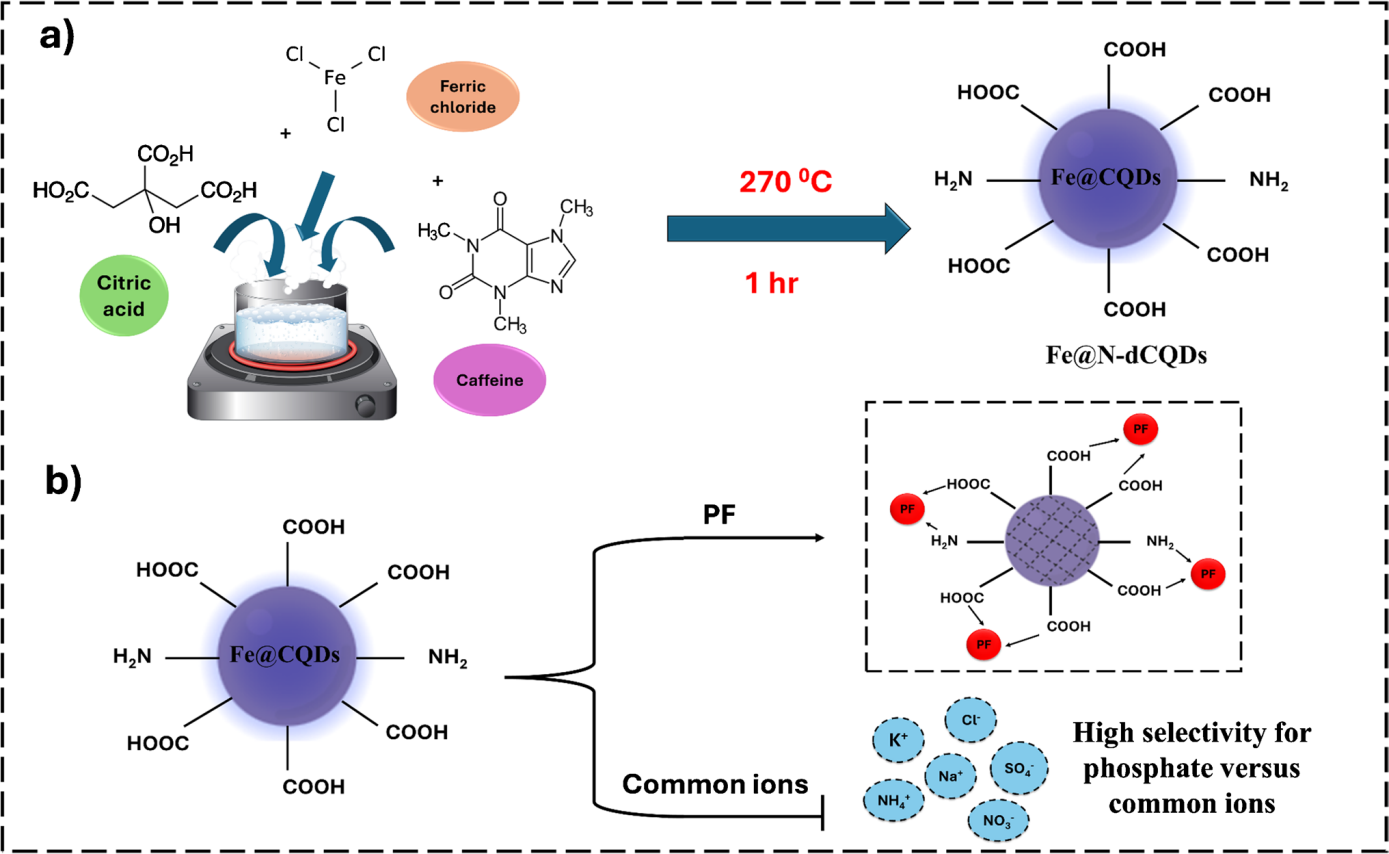
(**a**) The one-step pyrolytic synthesis of the Fe@N-dCQDs and (**b**) the mechanism for the detection of phosphate ions compared with common coexisting ions

Compared with previously reported CQD systems, the synthesized Fe@N-dCQDs differ in both composition and functionalization. Most CQDs in the literature are undoped or single-heteroatom doped, whereas the synthesized material features dual iron–nitrogen doping in a single-pot synthesis, creating abundant Fe–N catalytic centers not present in conventional CQDs. These structural and compositional attributes yield enhanced peroxidase-like activity, high substrate affinity, and improved stability, distinguishing the synthesized Fe@N-dCQDs from other carbon-based nanomaterials. A schematic illustration (Scheme 1) depicts the proposed mechanism and highlights how the Fe–N active centers selectively interact with PF ions. Pyrolysis also provides practical benefits over alternative synthesis methods, including simplicity, scalability, cost-effectiveness, ecological compliance, higher yields, faster processing, precursor versatility, and customizable optical/catalytic properties. All these advantages place pyrolysis as a preferable method for various applications, especially when sustainability, cost, and efficiency are of prior concern.

#### Characterizations of the synthesized Fe@N-dCQDs

**Fig. 1:**
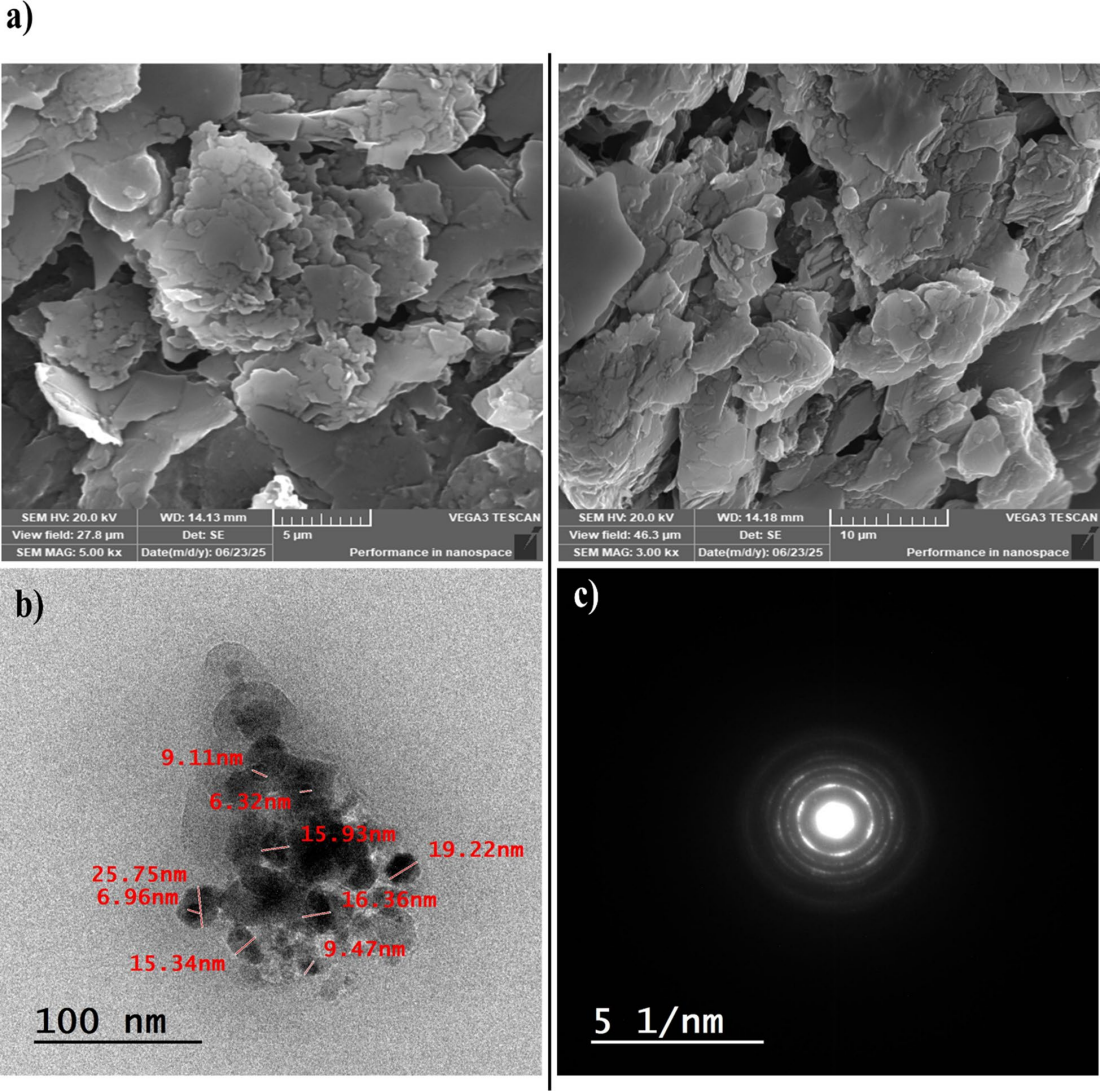
**a** The SEM images with different magnifications (5 µm and 10 µm), **b** the TEM analysis, and **c** the corresponding selected area electron diffraction (SAED) pattern of the synthesized Fe@N-dCQDs

Various techniques, including SEM, TEM, EDX, and FT-IR, were effectively employed for the identification and characterization of the synthesized QDs. SEM images offered detailed information regarding the morphology and surface texture of the synthesized QDs, showing aggregated, flaky structures with a rough and crumpled texture, ideal for catalytic applications (Fig. 1a). These layered flakes suggest a large surface area and porosity, which are advantageous for catalytic activity. Moreover, the morphology and particle size of the synthesized QDs were further examined using TEM (Fig. 1b). The images revealed that the QDs were nearly spherical with a tendency towards mild aggregation and particle sizes ranging from 6.0 to 26.0 nm, with D_10_, D_50_, and D_90_ values of 6.6, 15.3, and 23.9 nm, respectively, confirming the successful formation of nanosized particles within the quantum dot regime. Such morphology and size range are characteristic of CQDs synthesized via one-step pyrolysis, where instantaneous carbonization and Fe–N doping promote homogeneous nucleation with minimal aggregation. Similar size ranges have been reported for pyrolytically synthesized QDs exhibiting strong peroxidase-like catalytic activity [56–58]. The corresponding SAED pattern (Fig. 1c) exhibits multiple concentric diffraction rings with interplanar spacings rather than discrete spots, which are characteristic for a polycrystalline material. Together, the TEM and SAED results demonstrate that the synthetic route produced well-defined, polycrystalline iron quantum dots with high surface area and nanometer-scale dimensions suitable for catalytic or optoelectronic applications.

**Fig. 2:**
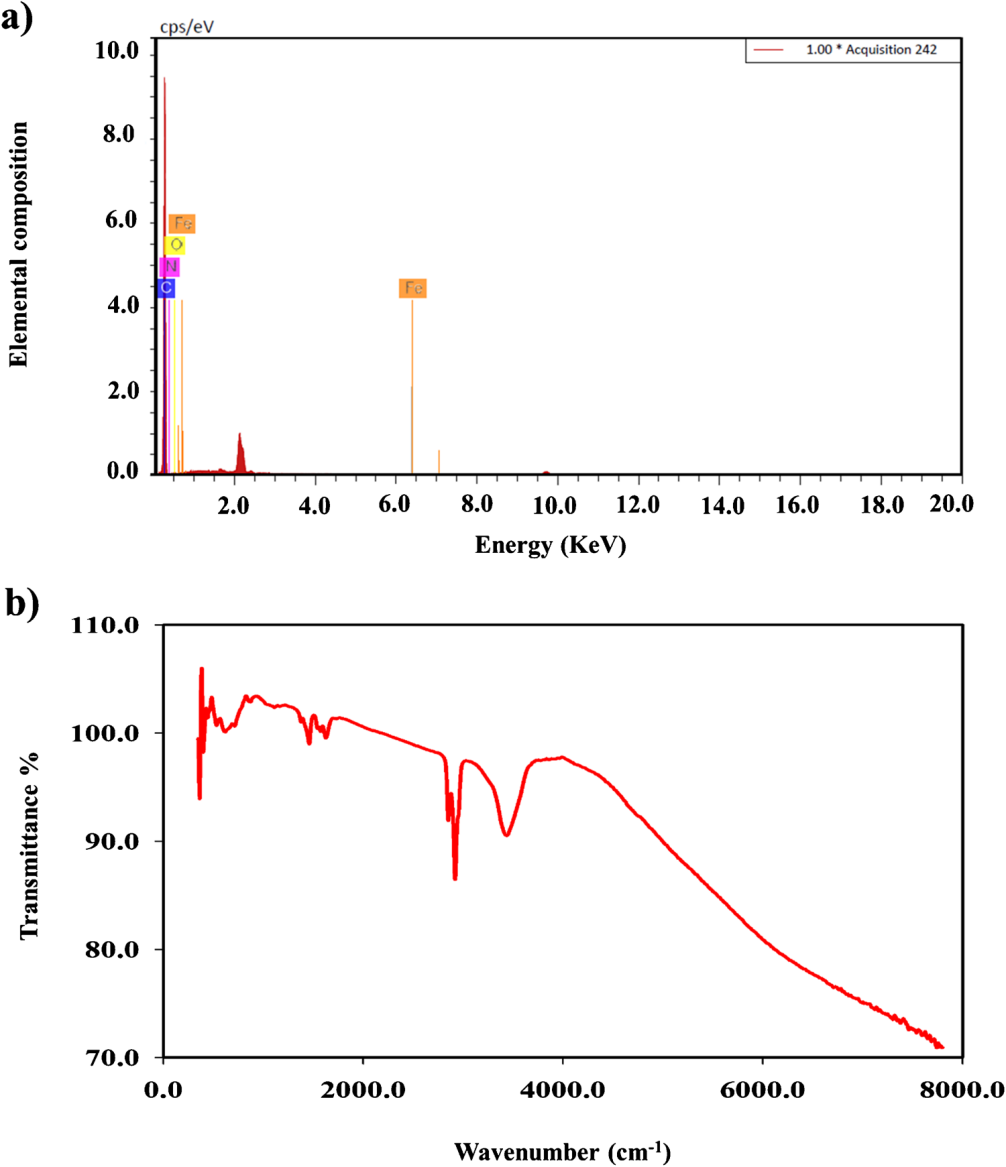
**a** The EDX elemental analysis and **b** the FT-IR spectra of the synthesized Fe@N-dCQDs

The EDX analysis further validated the elemental composition of the doped QDs, revealing competent integration and coexistence of C, N, O, and Fe elements, thereby indicating successful doping and surface functionalization (Fig. 2a). These structural and compositional features collectively support the dual-mode sensing performance observed in PF detection via both colorimetric and fluorometric pathways. Additionally, the FT-IR spectroscopy was employed to identify functional groups and surface modifications (Fig. 2b). A broad band around 3400 cm⁻^1^ was noticed and can be attributed to O–H or N–H stretching vibrations, indicating the presence of hydroxyl or amine groups on the nanoparticle surface that enhance water solubility (Fig. 2b). Peaks in the range of 2920–2850 cm⁻^1^ correspond to the C–H stretching of aliphatic hydrocarbons. Distinct absorption bands nearly at 1650 cm⁻^1^ and 1550 cm⁻^1^ are assigned to C=O (carbonyl) and C=C/C=N stretching, respectively, suggesting well graphitization and doping with nitrogen functionalities. A prominent peak around 1100 cm⁻^1^ likely results from C–N or Fe–O stretching, confirming successful nitrogen doping and iron incorporation. These findings not only validate the structural integrity of the QDs but also support their surface functionalization, chemical reactivity, and suitability in sensing, catalytic activities, and biological applications.

### Sensing system mechanism

During the current study, the synthesized Fe@N-dCQDs exhibited a dual functionality of possessing strong photoluminescence characteristics and peroxidase mimetic activity. The latter facilitates the colorimetric and chemiluminescent detection of analytes via catalytic oxidation reactions, while the intrinsic fluorescence allows for selective and sensitive signal transduction. These features were effectively employed for the selective sensing of PF ions. The detection mechanism relies on the PF-induced quenching of both the catalytic activity and intrinsic fluorescence of the synthesized Fe@N-dCQDs. This allows for rapid, label-free detection of PF with high sensitivity, good selectivity, and operational simplicity for environmental and biological monitoring applications.

#### Peroxidase mimetic activity of the Fe@N-dCQDs and PF inhibiting effect

**Fig. 3:**
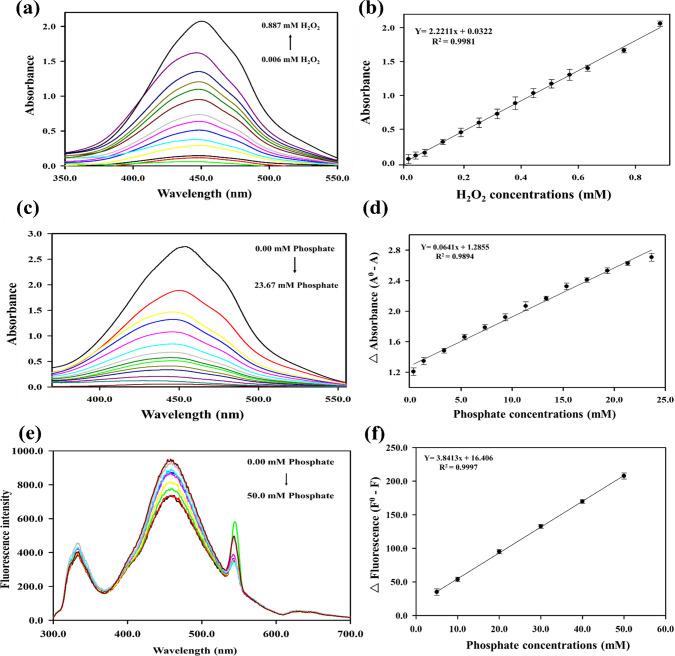
**a** The UV-visible absorption spectra and **b** calibration curve of hydrogen peroxide, **c** the UV-visible absorption spectra and **d** calibration curve of phosphate ion, **e** fluorescence quenching spectra, and **f** calibration curve of phosphate ions using the synthesized Fe@N-dCQDs as peroxidase mimetics

The catalytic reaction of the Fe@N-dCQDs towards OPD oxidation by H_2_O_2_ can serve as a reliable evaluator for their mimetic peroxidase activity. The synthesized Fe@N-dCQDs effectively catalyzed the oxidation of the OPD substrate upon using H_2_O_2_ as an oxidant at a temperature of 40 °C, giving a yellow-colored OPD_ox_ solution with a characteristic absorption spectrum at 448.4 nm. Notably, the absorption intensity was further increased upon using different concentrations of H_2_O_2_, emphasizing the catalytic performance and suitability of the synthesized Fe@N-dCQDs to be used as peroxidase analogs (Fig. 3a). However, upon introducing PF to the reaction system, the color intensity of the OPD_ox_ product diminishes, causing the absorbance intensities to decrease. Different PF concentrations (0.33–23.67 mM) were determined according to the change (decrease) in absorbance (Fig. 3c). This prevailing phenomenon may be attributed to the competing affinity of PF with the OPD substrate towards the active sites present on the Fe@N-dCQDs surface, altering their electronic structure, and thereby suppressing their catalytic activity. This causes the oxidation reaction to remarkably halt, consequently leading to a decrease in absorbance intensities. However, in the absence of PF, the catalytic activity of the synthesized Fe@N-dCQDs was enhanced. This inhibitory influence of PF can serve as a powerful indicator of its presence, leading to the feasible development of a more robust and accurate method for its quantification in various analytical fields.

#### Radical scavenging study

The catalytic mechanism of the synthesized Fe@N-dCQDs was further explored through a radical scavenging study. Isopropyl alcohol (IPA), a well-known hydroxyl radical quencher commonly applied as a spin-trapping agent in electron paramagnetic resonance (EPR) studies, was introduced into the OPD–H₂O₂ reaction system. Although EPR spin-trapping represents the most direct method for detecting transient radical species, IPA scavenging was utilized in this work as a practical mechanistic probe. The oxidation of OPD in the presence of H₂O₂ can proceed via two main pathways: (i) a radical-mediated route and (ii) direct surface electron transfer. Introducing IPA has led to a noticeable reduction in the absorbance of the oxidized OPD product (55% reduction) compared to the reaction without IPA. This inhibition is attributed to the trapping of hydroxyl radicals generated by the QDs, which decreases the availability of reactive species responsible for OPD oxidation. These observations highlight the crucial contribution of hydroxyl radicals in the catalytic oxidation of OPD and provide preliminary mechanistic evidence for ROS participation.

#### Fluorescence activity of the synthesized Fe@N-dCQDs and PF quenching mechanism

The fluorescence QY of the synthesized Fe@N-dCQDs was first determined using tryptophan as a reference fluorophore, yielding a high value of 0.885, which reflects the strong fluorescence efficiency of the prepared QDs. Such pronounced emission efficiency makes Fe@N-dCQDs highly suitable for sensitive fluorescence-based sensing applications.

**Fig. 4:**
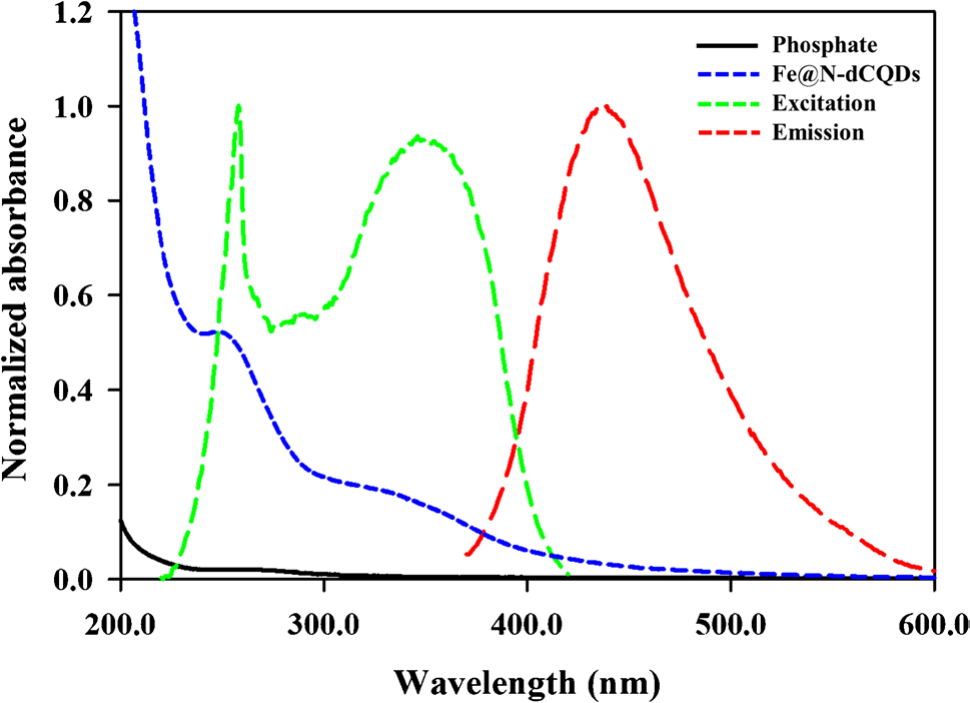
Normalized UV-vis absorbance of phosphate ions and the excitation/emission spectra of Fe@N-dCQDs. The minimal overlap between phosphate absorbance and the Fe@N-dCQDs excitation/emission regions confirms the absence of the inner filter effect (IFE)

There are different mechanisms by which Fe@N-dCQDs interact with pharmaceuticals or any analyte, including static quenching, dynamic quenching, and the inner filter effect (IFE). In the UV-visible spectrum, PF ions do not have notable light-absorbing features since their electronic structure does not have the transitions needed to interact with light in a meaningful way (Fig. 4). As a result, the optical behavior of Fe@N-dCQDs, particularly their excitation spectrum, remains unaffected in the presence of PF. Consequently, the IFE can be ruled out because there is no spectral overlap between the absorption of PF and the excitation/emission spectra of Fe@N-dCQDs (Fig. 4). To further confirm this, an integral overlap analysis was conducted to exclude the IFE, using the normalized absorption spectrum of PF ions with the excitation and emission spectra of the Fe@N-dCQDs. The overlap between the spectra was quantitatively assessed through the overlap integral as follows:

$${o}_{exc}=\frac{\int {A}_{PF(\lambda )} {E}_{\mathrm{Fe}@\mathrm{N}-\mathrm{dCQDs}\left(\lambda \right)} {d}_{\lambda }}{\int {E}_{\mathrm{Fe}@\mathrm{N}-\mathrm{dCQDs}\left(\lambda \right)} {d}_{\lambda }}$$ and $${o}_{em}=\frac{\int {A}_{PF(\lambda )} {F}_{\mathrm{Fe}@\mathrm{N}-\mathrm{dCQDs}\left(\lambda \right)} {d}_{\lambda }}{\int {F}_{\mathrm{Fe}@\mathrm{N}-\mathrm{dCQDs}\left(\lambda \right)} {d}_{\lambda }}$$

where *A*_*PF*(*λ*)_ is the absorbance spectrum of PF; *E*_Fe@N-dCQDs(λ)_ and *F*_Fe@N-dCQDs(λ)_ are the normalized excitation or emission intensity of the Fe@N-dCQDs at the *λ*_*max*_. The computed overlap integrals were 0.0094 and 0.0029 for *O*_*exc*_ and *O*_*em*,_ respectively, corresponding to less than 1% spectral overlap. The absorbances of PF at the excitation (258 nm) and emission (439 nm) maxima of Fe@N-dCQDs were only 0.020 and 0.003, respectively. These results confirm the negligible spectral interference between PF ions and Fe@N-dCQDs, indicating the absence of an IFE. Therefore, the fluorescence quenching observed in the sensing system is attributed to static complex formation rather than optical attenuation.

**Fig. 5:**
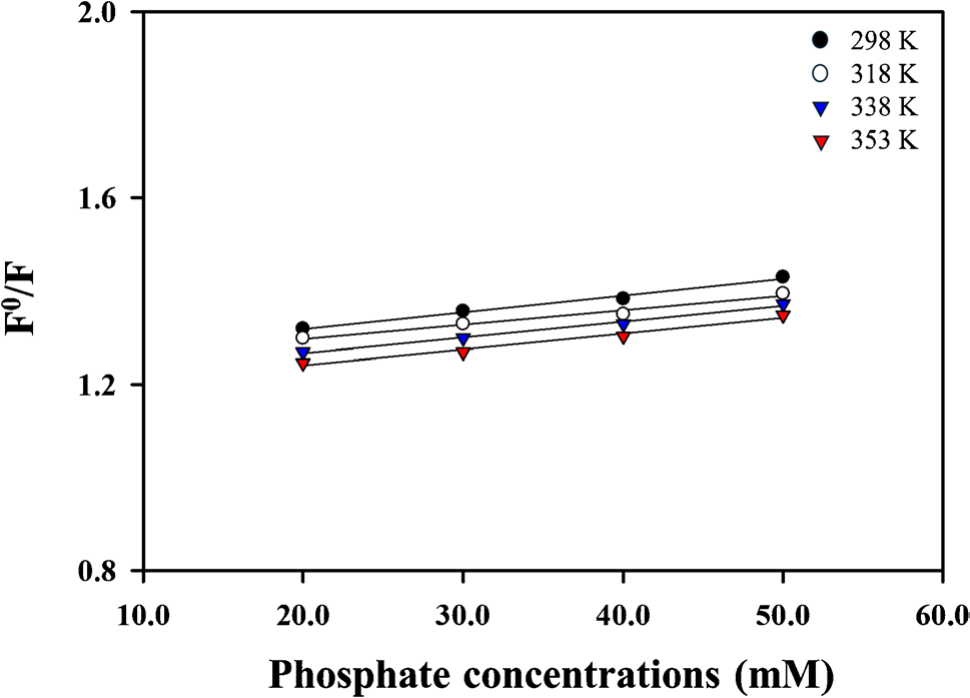
The quenching mechanism of phosphate ions using the synthesized Fe@N-dCQDs at different temperatures

The Stern-Volmer equation is used to figure out the static and dynamic quenching mechanisms, which are additional important mechanisms. The ground state complex formation causes static quenching, whereas the quencher (PF ions) travels in an excited state and interacts with the fluorophore, resulting in dynamic quenching. The two mechanisms differ depending on temperature, where increasing the temperature generates a drop in the Stern-Volmer constant (KSV) in static quenching and a rise in dynamic quenching. A possible quenching mechanism study was carried out using the Stern-Volmer formula: *F*^0^/*F* = 1 + *Ksv*[*Q*], where *Q*, *Ksv*, *F*^0^_,_ and *F* represent PF concentrations, the Stern-Volmer constant, the fluorescence intensities of the Fe@N-dCQDs in the absence of PF ions, and the fluorescence intensities of Fe@N-dCQD in the presence of PF ions, respectively. Using the Stern-Volmer equation, the suggested approach was implemented at different temperature settings (298 K, 318 K, 338 K, and 353 K). Figure 5 displays the curve of F^0^/F against the PF ions concentration. At temperatures of 298 K, 318 K, 338 K, and 353 K, respectively, the KSV values were 1.58 × 10^2^, 1.48 × 10^2^, 1.34 × 10^2^, and 1.25 × 10^2^. Based on the results obtained, the quenching mechanism is static quenching because of the KSV values’ apparent decrease with increasing temperature.

### Optimization of the experimental variables

#### Colorimetric determination

**Fig. 6:**
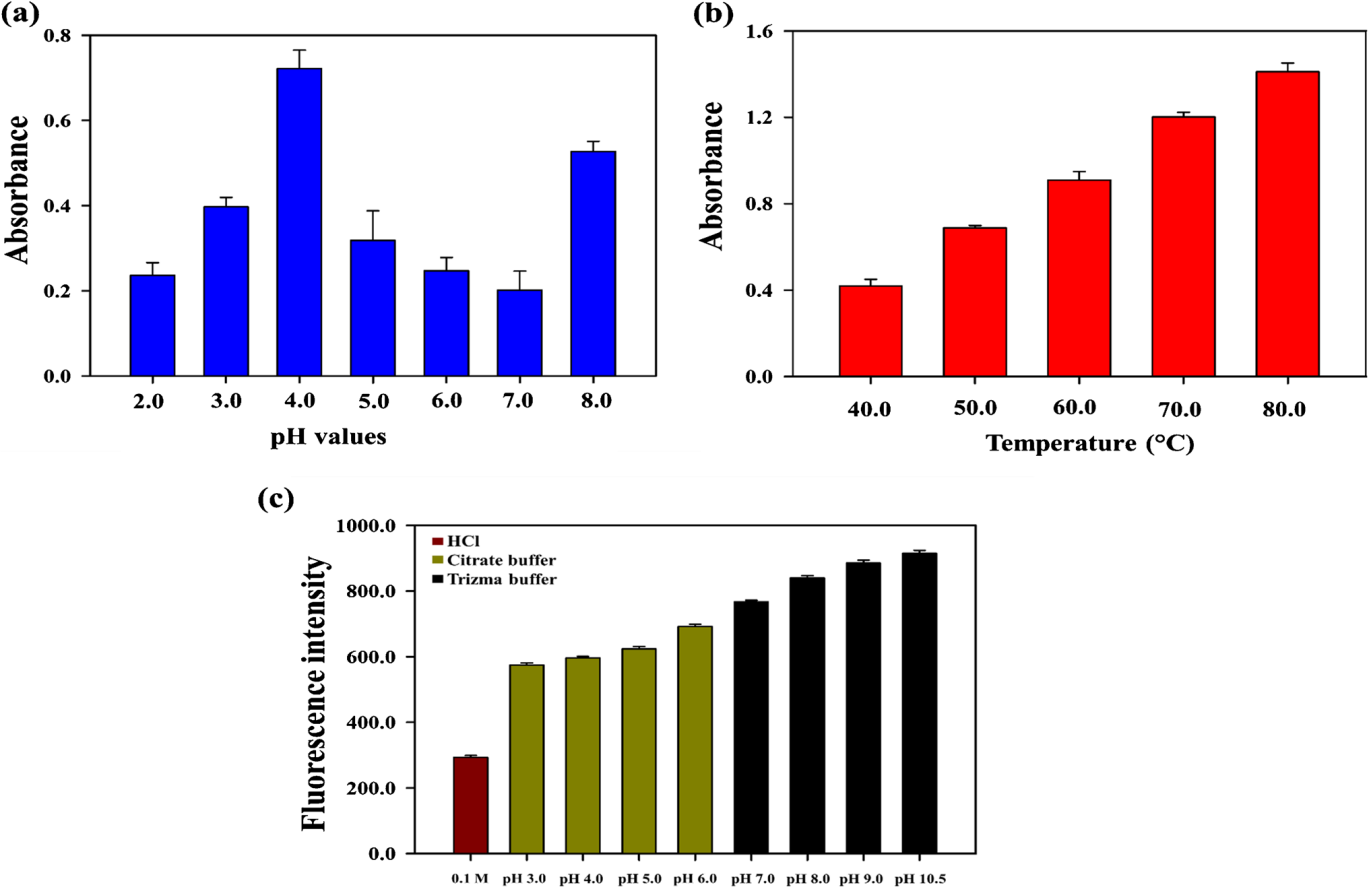
The effect of different **a** pH and **b** temperature values on the oxidation of OPD, while **c** represents the effect of pH values of different media on the fluorescence intensity of the synthesized Fe@N-dCQDs

The peroxidase-stimulating activity of the Fe@N-dCQDs was further assessed under varying conditions, including pH and temperature, to identify the optimal parameters. A range of pH values from 2.0 to 8.0 was tried to select the ideal medium. As shown in Fig. 6a, the catalytic activity of the synthesized QDs varied significantly with pH, where the highest activity was observed at a pH of 4.0, which was subsequently chosen for further experiments. Similarly, the effect of temperature was also evaluated to acquire the optimum one. Figure 6b indicates activity enhancement upon increasing the reaction temperature, and 40 °C was selected for conducting the catalytic reactions. While 40 °C may not represent the absolute optimal temperature, it was chosen to align with the objectives of developing an on-site point-of-care nanoprobe.

#### Fluorescence determination

The impact of pH values on the fluorescence intensity of the Fe@N-dCQDs in different media was evaluated. The results obtained revealed that at acidic pH values, the fluorescence intensity was low, and when the pH increased, a significant improvement in fluorescence intensity was observed. The optimal response was achieved at a pH of 10.5, suggesting that the electronic transitions that enhance fluorescence are more favorable in a basic environment (Fig. 6c). It was found that the medium at pH 10.5 facilitates the interaction between PF ions and Fe@N-dCQDs, which induces fluorescent changes enabling sensitive quantification. This study shows that the Fe@N-dCQDs’ optical properties are significantly affected by the pH value, which may have consequences for their use in sensing.

Additionally, the photostability of Fe@N-dCQDs was assessed by continuous illumination with a xenon lamp for 2 h, and the measured fluorescence intensities demonstrated excellent stability of Fe@N-dCQDs to photobleaching (see Electronic Supplementary Material Fig. S1a). The stability of Fe@N-dCQDs in high ionic strength solutions was investigated by measuring the fluorescence intensities after adding different high concentrations of NaCl solutions in the concentration range of 0.2 to 1.5 M. No notable changes in the fluorescence intensities were observed, demonstrating the stability of Fe@N-dCQDs in high ionic strength solutions (see Electronic Supplementary Material Fig. S1b).

Owing to its substantial impact on fluorescence intensity, the incubation period, which ranged from 2.0 min to 24.0 h, was evaluated after the addition of PF ions to the Fe@N-dCQDs. Notably, the system demonstrated excellent time efficiency as the fluorescence intensity was stable for at least 8.0 h, and the response between the studied analyte and the synthesized Fe@N-dCQDs was found to be immediate, taking only a few seconds to occur.

### Steady-state kinetics

The peroxidase mimetic property of the Fe@N-dCQDs was studied using steady-state kinetic assays with OPD as the chromogenic substrate. The catalytic behavior of the Fe@N-dCQDs followed typical MichaelisMenten kinetics, where the initial reaction rate increased progressively with higher OPD concentrations. Varied concentrations of OPD were employed during the study to produce the Michaelis-Menten curve and Lineweaver-Burk double-reciprocal model (see Electronic Supplementary Material Fig. S2). Initial reaction rates (**V₀**) were determined from the linear portion of the time absorbance traces and fitted to the Michaelis-Menten equation using nonlinear regression. Each substrate concentration was measured in triplicate, and values are reported as mean ± SD. Key kinetic parameters, including *K*_*m*_ and *V*_*max*_, were calculated, where *K*_*m*_ reflects the binding affinity of the substrate towards the nanozyme and *V*_*max*_ represents the maximum reaction rate, highlighting the catalytic efficiency and simulated enzymatic strength of the Fe@N-dCQDs. The calculated values of *K*_*m*_ and *V*_*max*_ of the synthesized Fe@N-dCQDs were 0.406 mM and 304.1 × 10^–8^ M S^−1^, respectively. Compared with other peroxidase mimetics, the *K*_*m*_ value was much lower than horseradish peroxidase (HRP, *K*_*m*_ = 1.80 mM, *V*_*max*_ = 0.12 × 10^–8^ M s^−1^), indicating the strong affinity of the OPD substrate towards the Fe@N-dCQDs (see Electronic Supplementary Material Table S1).

### Calibration curve construction and detection limit estimation

#### Peroxidase mimetic activity determination

The Fe@N-dCQDs activity was initially assessed at various concentrations of H_2_O_2_ to determine the dynamic range of the proposed method. Under optimized conditions, the absorption rates increased across the concentration range of 0.006 to 0.887 mM for H_2_O_2_ (Fig. 3b). A linear calibration curve was obtained within this range, yielding a detection limit of 0.003 mM and a correlation coefficient (*r*) of 0.9990. All measurements were performed in at least triplicate to ensure accuracy and reproducibility. For each concentration point, independent parallel experiments were carried out under identical conditions, and the mean values were used for plotting. Error bars shown in all figures represent the standard deviation (± SD) calculated from these replicate measurements. This approach helped to assess the variability of the data, verify the precision of the analytical method, and ensure the robustness of the reported calibration curves and kinetic parameters.

#### Phosphate determination

As previously described, the detection of PF mainly relies on significant changes in both absorbance and fluorescence intensities. In the presence of PF, both the catalytic activity and fluorescence intensity of the Fe@N-dCQDs are inhibited, resulting in a measurable decrease in absorbance and fluorescence values. Using the established conditions, a series of standard PF solutions with varying concentrations was successfully analyzed, and their related absorption spectra (Fig. 3c) and fluorescence spectra (Fig. 3e) were recorded. A standard curve was structured by plotting the absorbance differences (△A) against PF concentrations, demonstrating a direct linear relationship in the range of 0.33 to 23.67 mM with a limit of detection of 0.13 mM and an *r* of 0.9947 (Fig. 3d). However, regarding fluorescence measurements, the standard curve was established as a relationship between the fluorescence difference (△F) and PF concentrations (Fig. 3f). The fluorescence difference (△F) showed a linear correlation to PF concentrations in a concentration range of 5.0–50.0 mM with a limit of detection and an *r* of 1.72 mM and 0.9998, respectively. Compared with the reported methods for PF determination, the developed method exhibited satisfactory sensitivity and linearity (see Electronic Supplementary Material Table S2), highlighting the robustness of the synthesized quantum dots towards diverse applications.

### Determination of phosphate in different matrices

To further validate the applicability of the developed nanozyme-based sensing system for real sample analysis, PF concentrations were determined in agricultural soil, river water, and three commercial fertilizer samples using the standard addition technique. Known concentrations of PF standards (3.33–13.33 mM) were spiked into the pre-treated samples and analyzed under the optimized assay conditions. The quenching of the OPD oxidation signal increased proportionally with PF addition, allowing accurate quantification of the native PF levels.

As shown in Table 1, excellent recoveries were obtained, ranging from 95.20 to 98.04% for soil, 97.00 to 103.30% for river water, and 95.30 to 105.40% for fertilizer samples, confirming negligible matrix interferences. The fertilizers claimed to contain 20% PF (as P), and the measured values were consistent with the labeled content. Additionally, statistical comparison with the reference method using Student’s *t*-test and *F*-test demonstrated no significant difference at the 95% confidence level (Table 2), highlighting the accuracy and precision of the proposed method for practical applications in environmental and agricultural analysis. Overall, these results demonstrated the wide applicability of this sensing approach, offering high sensitivity, strong selectivity, and promising applicability for practical environmental monitoring.

**Table 1:** Phosphate determination in different matrices

Quantity of phosphate						Recovery % ± RSD*				
Added (mM)	Found (mM)									
	Soil	River	Fertilizer 1	Fertilizer 2	Fertilizer 3	Soil	River	Fertilizer 1	Fertilizer 2	Fertilizer 3
3.33	3.24	3.23	3.51	3.22	3.42	97.29 ± 1.882	96.99 ± 2.21	105.40 ± 1.03	96.69 ± 2.07	102.70 ± 1.99
6.66	6.53	6.76	6.65	7.02	6.74	98.04 ± 1.624	101.50 ± 1.44	99.84 ± 1.46	105.40 ± 1.74	101.20 ± 1.73
10.00	9.52	10.33	9.88	10.23	9.53	95.20 ± 2.036	103.30 ± 1.31	98.80 ± 1.89	102.30 ± 2.11	95.30 ± 2.32
13.33	12.88	12.95	13.69	13.26	13.11	96.62 ± 1.982	97.15 ± 2.33	102.70 ± 0.98	99.47 ± 1.52	98.35 ± 1.14

*Average of three determinations

**Table 2:** Statistical comparison between the proposed and a reference method for the determination of phosphate in different matrices

Parameters		Sample				
	Soil	River	Fertilizer 1	Fertilizer 2	Fertilizer 3	Reference method
**Mean**	96.79	99.74	101.69	100.97	99.39	99.88
**SD**	1.881	1.822	1.340	1.860	1.795	1.563
**Variance**	3.538	3.320	1.796	3.460	3.222	2.443
***n***	4					
**Student *t*-test **^**a**^**(2.776)**	2.527	0.117	1.758	0.897	0.412	
***F* value **^**a**^**(9.28)**	0.690	1.359	0.735	1.416	1.319	

^a^The values in parentheses are the corresponding tabulated two-tailed values at *p* = 0.05

### Stability and phosphate interaction

The Fe@N-dCQDs showed excellent fluorescence and catalytic stability during the detection process. The apparent decrease in fluorescence intensity upon PF addition is attributed to the strong binding of PF ions to the iron active sites, which suppresses the peroxidase-like activity without altering the structural integrity of the nanocomposite. This inhibition is reversible and does not involve degradation of the CDs’ framework. The consistent emission and catalytic responses throughout the experiments further indicate that the material remains stable under the applied conditions. These findings agree with previous studies reporting that PF species can temporarily deactivate Fe-based nanozymes without altering their morphology or composition [59].

### Selectivity study

**Fig. 7:**
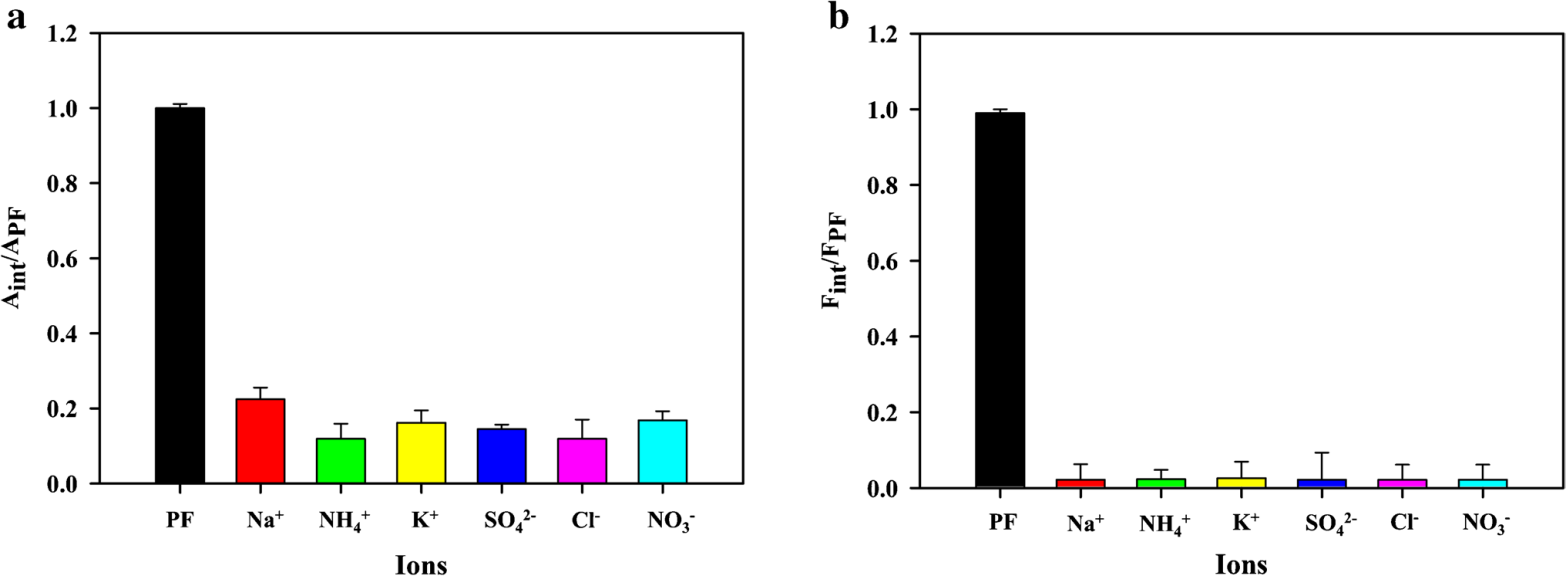
Influences of various interfering substances on the **a** colorimetric and **b** fluorimetric detection systems

The method’s specificity towards PF detection was evaluated through the incorporation of several ions, commonly present in environmental matrices, including phosphatic fertilizers, into the reaction mixture as interfering substances. Ions such as Na^+^, NH_4_^+^, K^+^, SO_4_^2−^, Cl^−^, and NO_3_^−^ ions were selected as a test study. A concentration of 16.60 mM was used for the interfering substances, whereas that of PF was 1.66 mM, which is 10 times less than the interfering ions. As shown in Fig. 7a, the peroxidase mimetic activity and the fluorescence intensity (Fig. 7b) of the synthesized Fe@N-dCQDs remained unaffected by the interferants, comparable to PF activity towards the QDs.

This selectivity can be explained by the strong affinity of PF ions for the Fe^3^⁺/Fe^2^⁺ coordination sites on the Fe@N-dCQDs, which favors their adsorption or complexation over other competing anions. PF forms stable inner-sphere complexes with the Fe–N catalytic sites. The nitrogen atoms introduced from caffeine and the carboxylate groups from citric acid serve as anchoring ligands surrounding the iron atoms, generating a dense network of Fe–N coordination centers. These centers function as preferential binding sites for PF, allowing it to replace weakly bound surface species and establish Fe–O–P linkages or hydrogen-bonded adducts. Such interactions not only enhance adsorption but also stabilize the transition state in the peroxidase-like reaction, intensifying the observed signal response. In contrast, other ions such as Na⁺, NH₄⁺, K⁺, SO₄^2^⁻, Cl⁻, and NO₃⁻ exhibit much weaker binding to iron, rendering them less competitive under identical conditions. Moreover, the tetrahedral structure of PF and its multiple oxygen donor atoms enable chelation or bridging between adjacent Fe centers on the quantum dot surface, an ability lacking in simpler anions. This chelation strengthens binding and enhances electronic coupling with Fe–N sites, amplifying the catalytic inhibition and fluorescence quenching effects.

Altogether, these structural and chemical factors elucidate why PF uniquely interacts with Fe@N-dCQDs to induce pronounced catalytic and optical responses, whereas other anions show negligible effects even at tenfold higher concentrations. The results highlight the robustness and selectivity of the sensor, confirming its suitability for accurate PF detection in complex matrices such as agricultural and fertilizer samples.

## Conclusion

Detecting PF levels is essential due to their environmental, biomedical, and agricultural importance. In the current study, a dual-mode sensing strategy was developed for assaying PF ions based on using the Fe@N-dCQDs nanozymes. The synthesized nanozymes exhibited strong photoluminescence and intrinsic peroxidase-like activity, permitting both fluorescence and colorimetric detection mechanisms. However, the presence of PF ions induced significant quenching in both the fluorescence intensity and catalytic activity of the Fe@N-dCQDs, enabling a sensitive and label-free detection strategy. The method showed excellent linearity across relevant PF concentration ranges in both detection modes, achieving low limits of detection and high correlation coefficients. The developed sensor was able to effectively determine PF concentrations in different environmental matrices, including agricultural soil and river water, along with commercially available fertilizers, indicating good applicability for environmental monitoring. Additionally, the sensor showed high selectivity for PF ions over various potential interfering species such as Na^+^, NH_4_^+^, K^+^, and others. Owing to its simplicity, rapid response, and dual-detection capability, the developed system presents a promising tool for environmental monitoring of PF in agricultural, biomedical, and environmental aspects. The integration of quantum dot–based optical sensing with enzyme-mimetic catalysis provides a powerful and versatile platform that can be extended to other analytes in future applications.

## Supplementary Information

Below is the link to the electronic supplementary material.

Supplementary Material 1 (DOCX 115 KB)

## Data Availability

All relevant data used in this study are available from the corresponding author upon reasonable request.
