# Supplementary material for: Dual-mode sensing strategy for assaying phosphate ions using Fe, N-co-doped carbon dots with peroxidase mimetic activity
Source: Anal Bioanal Chem. 2025 Dec 26;418(4):1259–75. doi: 10.1007/s00216-025-06279-z (PMC12901204; doi:10.1007/s00216-025-06279-z)
Supplement: Supplementary file 1 — Supplementary Material 1 (DOCX 115 KB) [file 216_2025_6279_MOESM1_ESM.docx]

**Supplementary Materials of**

**Dual-Mode Sensing Strategy for Assaying Phosphate Ions Using Fe, N-co-doped Carbon Dots with Peroxidase-Mimetic Activity**

Shymaa S. Soliman ^a,1*^, Amr M. Mahmoud ^b,2*^, Amira M. Kessiba ^c,3^, Rasha M. Ahmed ^c,4^

^a^ Analytical Chemistry Department, Faculty of Pharmacy, October 6 University, October 6 City, Giza, 12858, Egypt.

^b^ Pharmaceutical Analytical Chemistry Department, Faculty of Pharmacy, Cairo University, El-Kasr-El Aini Street, Cairo, 11562, Egypt.

^c^ Pharmaceutical Chemistry Department, Faculty of Pharmacy, Misr International University, Misr-Ismailia Road, Cairo, 4650241, Egypt.

* Corresponding authors' emails:

^a^ [shimaasayed@o6u.edu.eg](mailto:shimaasayed@o6u.edu.eg)

^b^ amr.bekhet@pharma.cu.edu.eg

* Mailing address:

^a^ Analytical Chemistry Department, Faculty of Pharmacy, October 6 University, October 6 City, Giza, 12858, Egypt.

^b^ Pharmaceutical Analytical Chemistry Department, Faculty of Pharmacy, Cairo University, El-Kasr-El Aini Street, Cairo, 11562, Egypt.

* Tel:

^a^ +20-1004581866 and ^b^ +20-1018338306

^1^ ORCID ID: 0000-0002-0554-0423

^2^ ORCID ID: 0000-0002-7804-6442

^3^ ORCID ID: 0000-0002-9655-976X

^4^ ORCID ID: 0000-0001-8256-6582


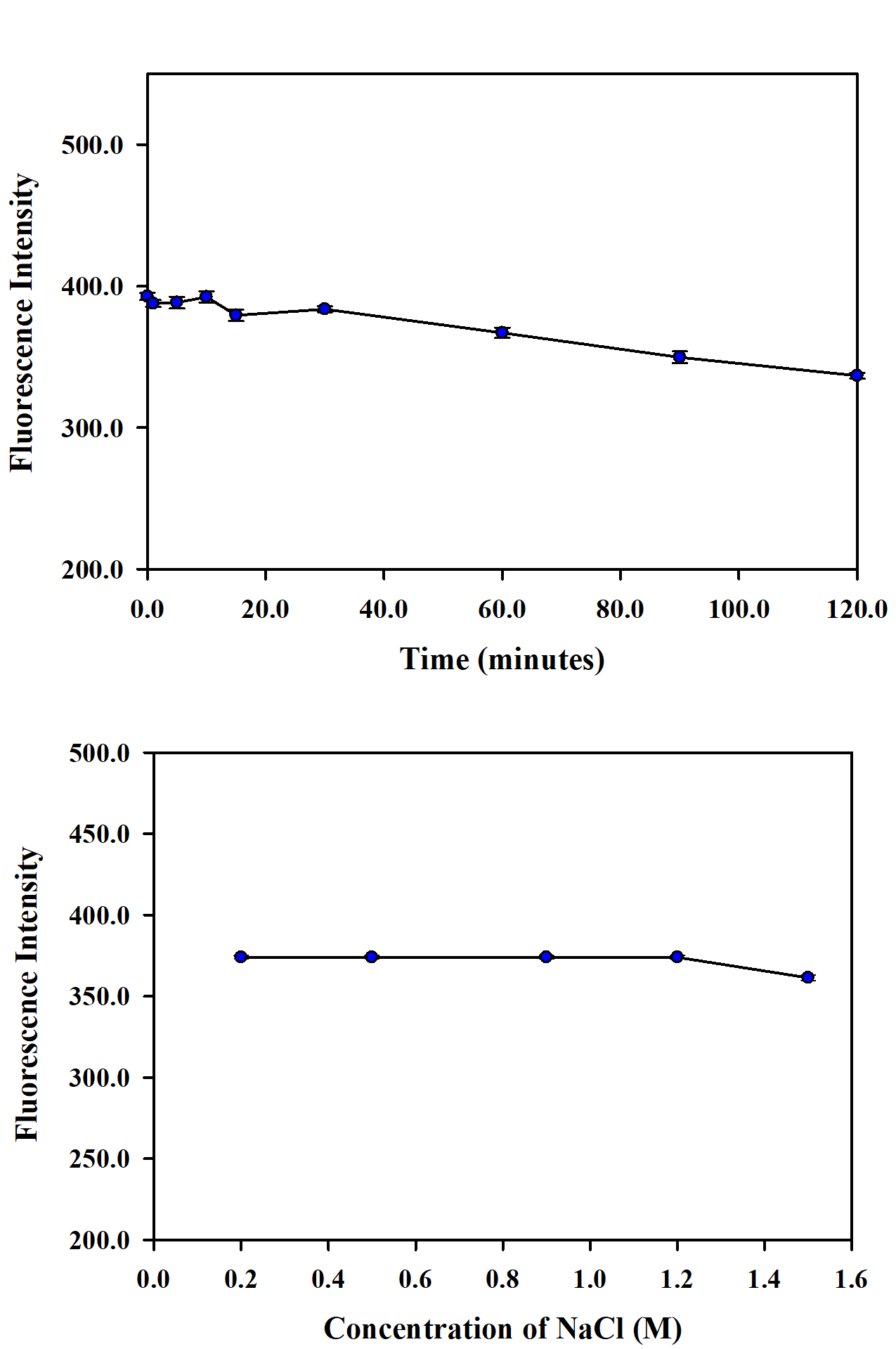


**Supplementary Figure S1.** The effect of **(a)** illumination time and **(b)** different concentrations of NaCl on the fluorescence intensity of the synthesized Fe@N-dCQDs

**
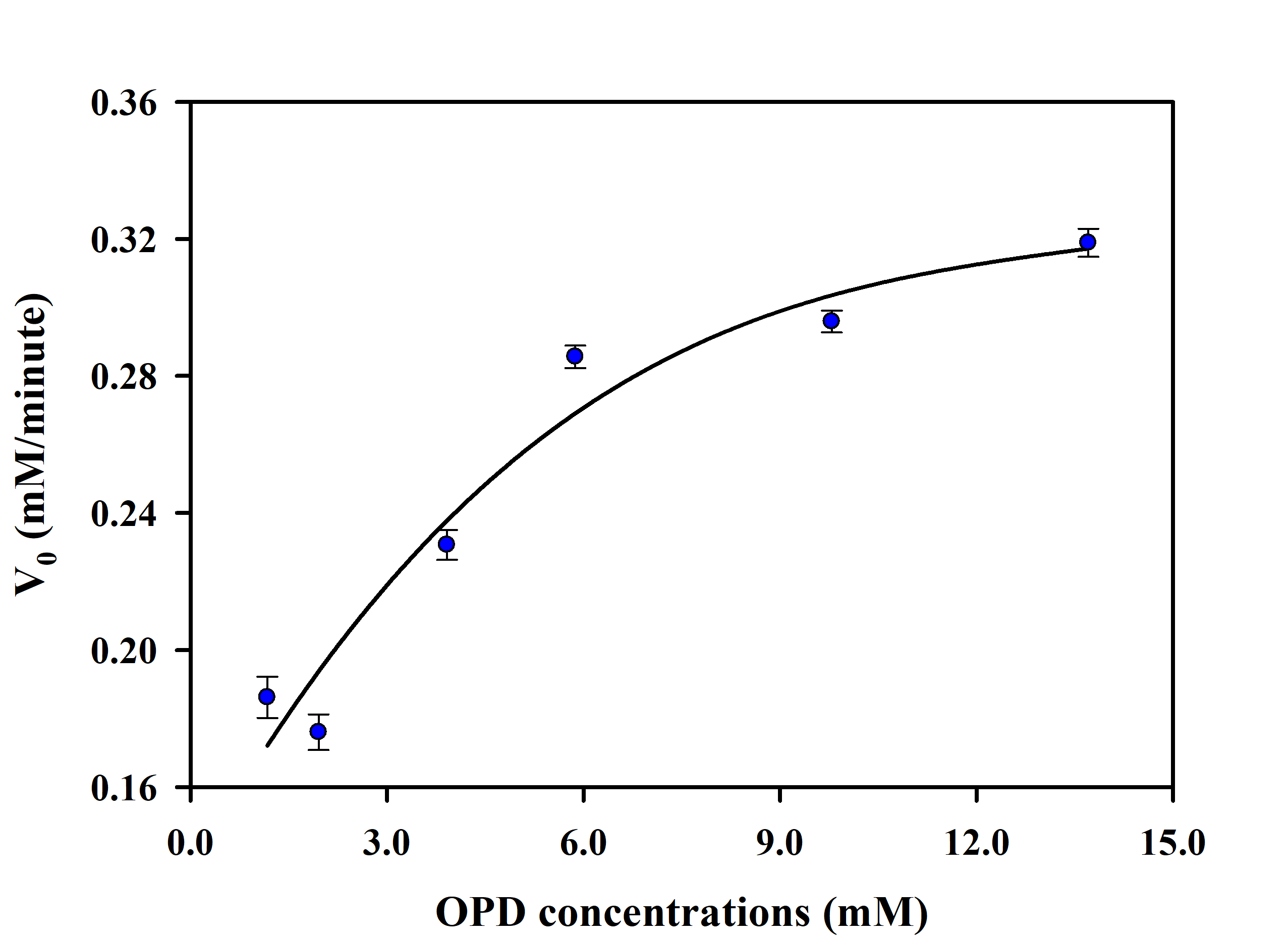
**

**Supplementary Figure S2.** Steady-state kinetics represent Michaelis-Menten curve for the reaction of OPD with fixed concentration of H_2_O_2_ in the present of Fe@N-dCQDs nanozymes

**Supplementary Table S1.** Study of the oxidation kinetic parameters of OPD using Fe@N-dCQDs nanozymes compared to other reported quantum dot nanomaterials.

| **Catalyst** | **K_m_ (mM)** | **V_max_ (10^-8^ M S^-1^)** | **Reference** |
| --- | --- | --- | --- |
| HRP ^*^ | 1.80 | 0.12 | (1) |
| Cu-CDs/Fe^2+^ | 0.64 | 4.96 | (2) |
| Cu-QDs NPs | 14.27 | 233.16 | (3) |
| CuFe-CQDs | 0.595 | 3.69 | (4) |
| Co-CDs | 7.08 | 8.64 | (5) |
| Fe@N-dCQDs | 0.406 | 304.09 | This study |

^*^The HRP kinetic parameters are cited from literature, obtained under phosphate buffer pH 6.0 with 2.5 × 10⁻⁴ mol L⁻¹ o-phenylenediamine and 1.25 × 10⁻⁴ mol L⁻¹ H₂O₂ (60 s reaction time). These values are provided solely for qualitative comparison with our catalyst.

**Supplementary Table S2.** Studying the sensing performance of the proposed colorimetric method for H_2_O_2_ determination using OPD substrate and Fe@N-dCQDs nanozymes compared to other reported quantum dots.

|  | **Colorimetry** | | **Fluorimetry** | |  |
| --- | --- | --- | --- | --- | --- |
| **Catalyst** | **Linearity range (mM)** | **Detection limit (mM)** | **Linearity range (mM)** | **Detection limit (mM)** | **Ref.** |
| Cu-CDs/Fe^2+^ | ----^*^ | ----^*^ | 0.0002 – 0.1 | 0.000036 | (2) |
| Cu-QDs NPs | 0.04 – 0.15 | ----^*^ | ----^*^ | ----^*^ | (3) |
| CuFe-CQDs | 0.0001 – 1.00 | 0.000019 | 0.001 – 1.0 | 0.000146 | (4) |
| Co-CDs | ----^*^ | ----^*^ | 0.5 – 150 | 0.1 | (5) |
| N/Cl-CDs | ----^*^ | ----^*^ | 0.001 – 0.03 | 0.0002 | (6) |
| Ce^4+^-GQDs | ----^*^ | ----^*^ | 0.001 – 0.1 | 0.00003 | (7) |
| Fe@N-dCQDs | 0.03 – 0.57 | 0.02 | ---- | ---- | This study |

^*^ The sign “----” means that the data was not provided in the literature.

**References:**

1. Kergaravat S V., Pividori MI, Hernandez SR. Evaluation of seven cosubstrates in the quantification of horseradish peroxidase enzyme by square wave voltammetry. Talanta. 2012;88:468–76. Available from: https://www.sciencedirect.com/science/article/abs/pii/S0039914011009945

2. Yang D, Li Q, Tammina SK, Gao Z, Yang Y. Cu-CDs/H2O2 system with peroxidase-like activities at neutral pH for the co-catalytic oxidation of o-phenylenediamine and inhibition of catalytic activity by Cr(III). Sensors Actuators B Chem. 2020;319:128273. Available from: https://sci-hub.se/https://doi.org/10.1016/j.snb.2020.128273

3. Thiyam DS, Nongmeikapam AC, Nandeibam AD, Heikham FD, Henam PS. Biosynthesized Quantum Dot Size Cu Nanocatalyst: Peroxidase Mimetic and Aqueous Phase Conversion of Fructose. ChemistrySelect. 2018;3(43):12183–91. Available from: /doi/pdf/10.1002/slct.201802236

4. Wang J, Yu G, Wu S, Zhou H, Liu X, Han J, et al. Bimetallic-doped carbon quantum dots with enhanced photoluminescence and peroxidase-mimicking activity for dual-mode colorimetric and ratiometric fluorescence assay of alendronate. Anal Bioanal Chem. 2025;1–12. Available from: https://link.springer.com/article/10.1007/s00216-025-05866-4

5. Guo J, Zhang J, Tong X. Adhered-3D paper microfluidic analytical device based on oxidase-mimicking activity of Co-doped carbon dots nanozyme for point-of-care testing of alkaline phosphatase. Anal Chim Acta. 2024;1332:343378. Available from: https://pdf.sciencedirectassets.com/271374/1-s2.0-S0003267024X00458/1-s2.0-S0003267024011796/main.pdf?X-Amz-Security-Token=IQoJb3JpZ2luX2VjELn%2F%2F%2F%2F%2F%2F%2F%2F%2F%2FwEaCXVzLWVhc3QtMSJHMEUCIBfvS0grWx7Dq6lL7n6UUzzKBgSPUEA2RbRykoQM%2Fo8WAiEA3GW%2F2%2B

6. Mathivanan D, Tammina SK, Wang X, Yang Y. Dual emission carbon dots as enzyme mimics and fluorescent probes for the determination of o-phenylenediamine and hydrogen peroxide. Microchim Acta. 2020;187(5):292. Available from: https://link.springer.com/article/10.1007/s00604-020-04256-0

7. Xia M, Zhao X-E, Sun J, Zheng Z, Zhu S. Graphene quantum dots combined with the oxidase-mimicking activity of Ce4+ for ratiometric fluorescent detection of Ce4+ and alendronate sodium. Sensors Actuators B Chem. 2020;319:128321. Available from: https://www.sciencedirect.com/science/article/abs/pii/S0925400520306663
